# SARS‐CoV‐2 vaccine‐triggered conversion from systemic lupus erythematosus (SLE) to bullous SLE and dipeptidyl peptidase 4 inhibitors‐associated bullous pemphigoid

**DOI:** 10.1111/1346-8138.16687

**Published:** 2022-12-28

**Authors:** Yukiko Nakahara, Mariko Yamane, Midori Sunada, Yumi Aoyama

**Affiliations:** ^1^ Department of Dermatology Kawasaki Medical School Okayama Japan

**Keywords:** bullous, systemic lupus erythematosus bullous pemphigoid, COVID‐19 vaccination, dipeptidyl peptidase‐4 inhibitor, systemic lupus erythematosus

## Abstract

Bullous systemic lupus erythematosus (BSLE) is a rare blistering disease in patients with SLE. BSLE is a heterogenous disease caused by autoantibodies to the basement membrane, mainly type VII collagen. The pathogenesis of the development of autoantibodies in BSLE remains unknown. We report a case of SLE taking dipeptidyl peptidase 4 inhibitors (DPP4i) who developed tense blister lesions after administration of SARS‐CoV‐2 vaccine. Initial erythematous lesion before administration of SARS‐CoV‐2 vaccine had not shown IgG deposition at basement membrane both direct and indirect immunofluorescence (IIF). However, the result of those examinations became positive after the administration of SARS‐CoV‐2 vaccine. Furthermore, IIF test results using NaCl split skin had shown positive against epidermal side. These observations suggest that SARS‐CoV‐2 vaccination triggered production of autoantibodies that cause bullous SLE. The present case fulfills the diagnostic criteria for both BSLE and DPP4i‐associated bullous pemphigoid. Skin lesions were cleared after withdrawal of DPP4i. Therefore, physicians should ask patients who develop blisters after the vaccination whether they are taking DPP4i.

## 1 INTRODUCTION

Systemic lupus erythematosus (SLE) and bullous pemphigoid (BP) are chronic autoimmune diseases in which autoantibodies (auto‐Abs) with different specificities are implicated in the induction and progression of these diseases. A few cases of coexistent SLE and BP or conversion from SLE to BP have been reported in the literature.^1^, ^2^ It has recently been reported that SARS‐CoV‐2 mRNA vaccines could trigger the development of new‐onset BP or BP flares in a patient with bullous LE^3^, ^4^ while both new‐onset and flares of SLE have been also observed after the inoculation of SARS‐CoV‐2 vaccine.^5^, ^6^ However, no case of new‐onset dipeptidyl peptidase 4 inhibitors‐associated BP (DPP‐4i‐BP) occurring after SARS‐CoV‐2 mRNA vaccination in patients with SLE has been reported. In considering that more severe COVID‐19 outcomes in individuals with SLE are largely driven by demographic factors, comorbidities, male gender, age, and untreated or active SLE, we raised concerns over adverse events occurring after vaccination in an elder, male patient with diabetes mellitus and bullous SLE. In this report, we describe the case of new‐onset DPP‐4i‐BP following SARS‐CoV‐2 mRNA vaccination, which would serve to unforeseen conversion from SLE to bullous SLE. This case report may encourage further clinical studies on the role of SARS‐CoV‐2 mRNA vaccination on the conversion of autoimmune disease.

## 2 CASE PRESENTATION

A 71‐year‐old‐man with type 2 diabetes mellitus presented with blistering lesions on the neck and wrist. His medical history included hemophagocytic lymphohistiocytosis (HLH). One year earlier he had developed fever, pancytopenia, hepatosplenomegaly, and hyperferritinemia, referred to as HLH without any triggering factors. He was receiving pulse corticosteroids twice, leading to a progressive improvement of HLH.

**FIGURE 1:**
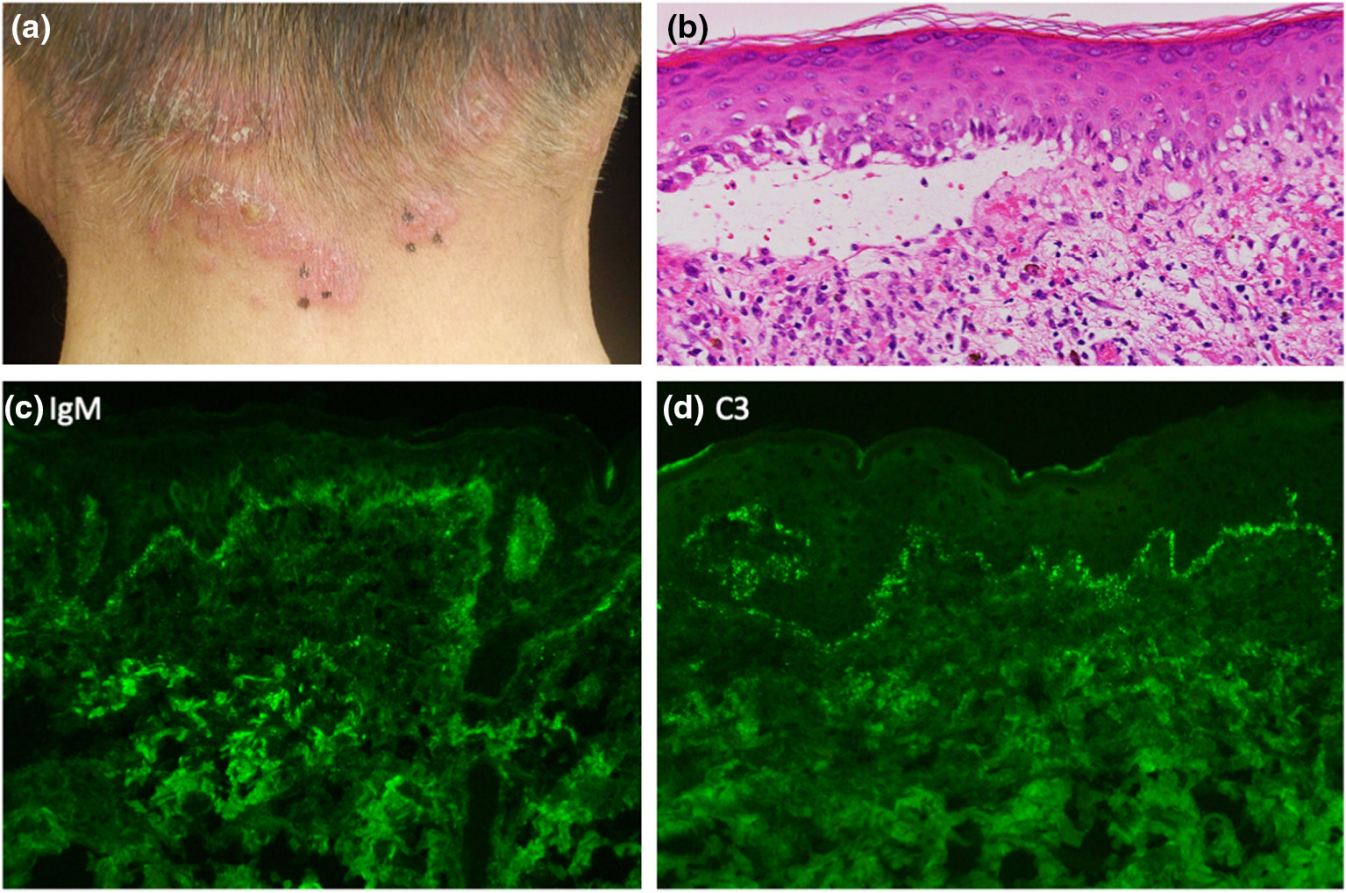
Clinical presentation of case1 before SARS‐CoV‐2 vaccination. (a) Erythema with crust on the neck. (b) Subepidermal cleft with infiltration of lymphocytes. ×400. (c) IgM deposition in direct immunofluorescence. ×400. (d) C3 deposit in direct immunofluorescence. ×400.

**FIGURE 2:**
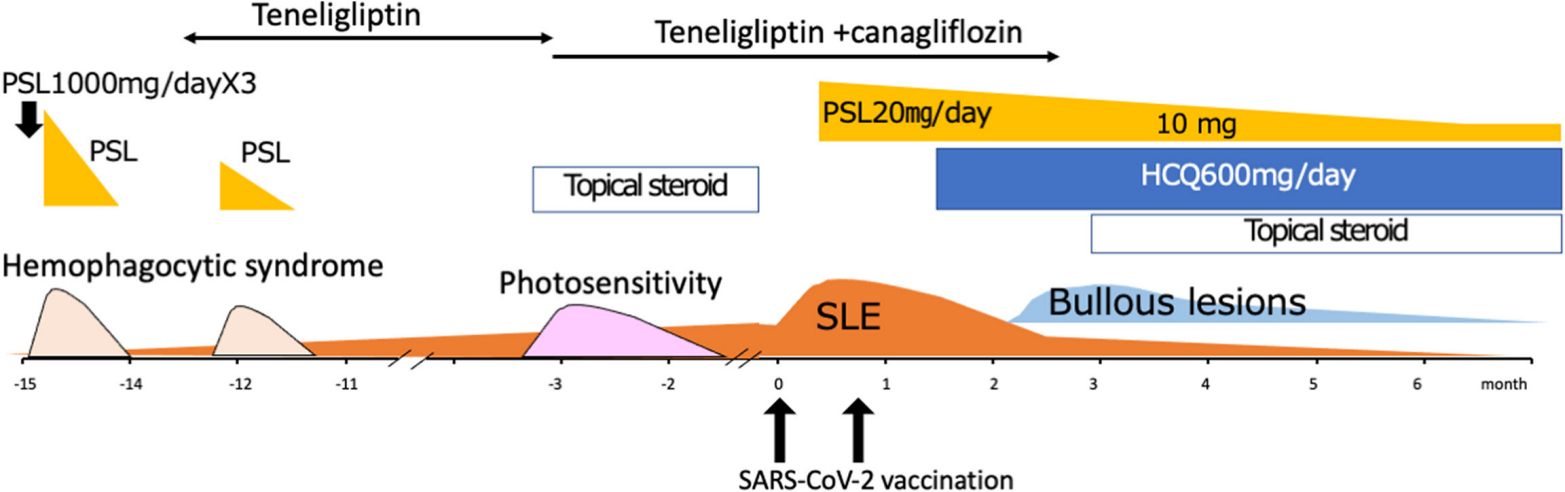
The chronology of symptomatic manifestations and treatment during the clinical course of case 1. HCQ, hydroxychloroquine; PSL, predonisone, SLE, systemic lupus erythematosus.

**FIGURE 3:**
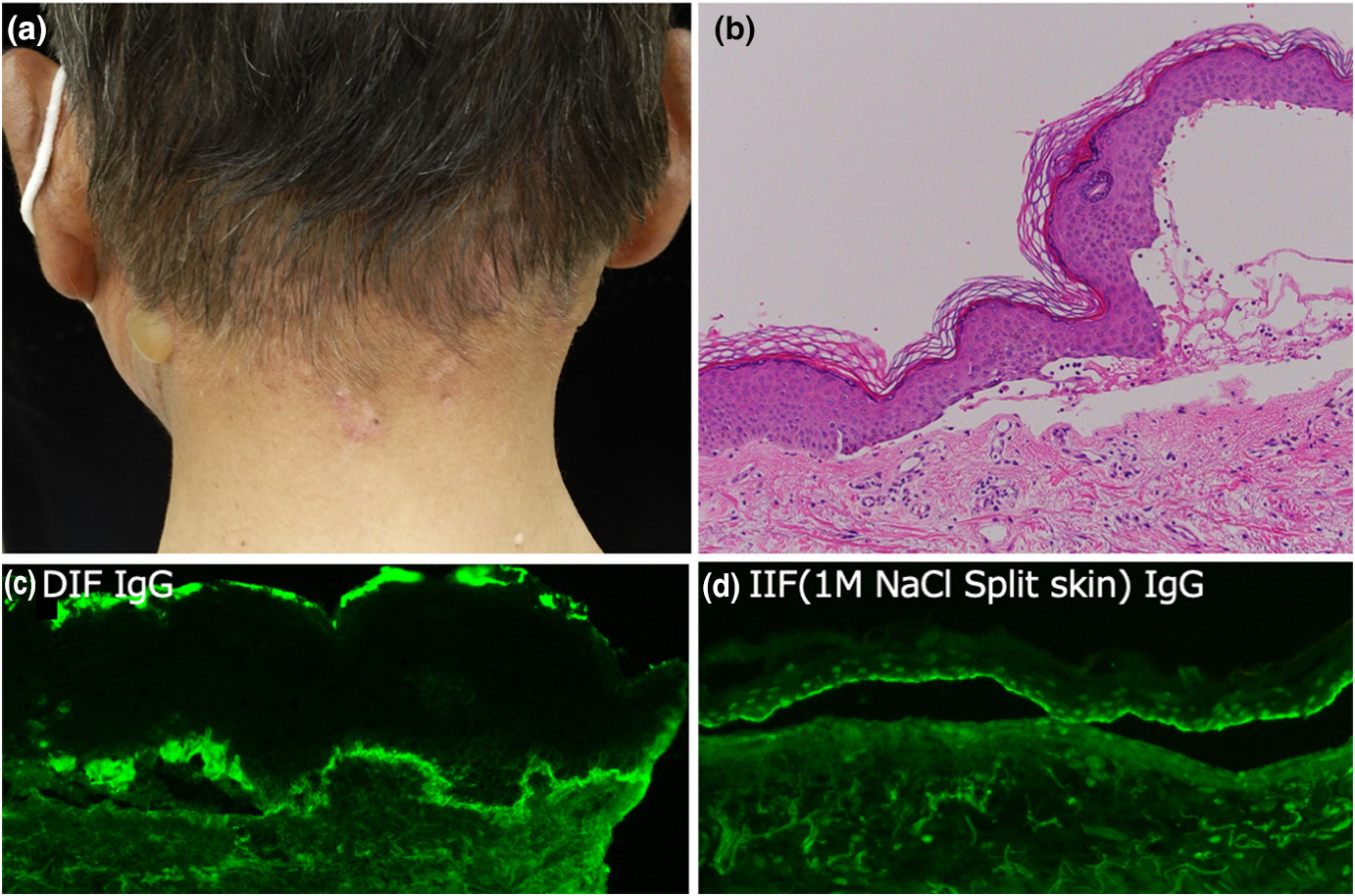
SARS‐CoV‐2 vaccination triggered blister in case1. (a) Tense bulla at the neck. (b) HE staining showing subepidermal blister. ×200. (c) IgG deposition in indirect immunofluorescence. ×400. (d) IgG deposition at the epidermal side of 1 M NaCl salt‐spilt skin. ×400.

Physical examination showed erythematous plaques with crusting lesions over the face and sun‐exposed areas of neck, palm, and fingers (Figure 1a). Laboratory tests revealed a white blood cell count of 3750/μL, hemoglobin of 12.5 g/dL, and platelet count of 12.2 × 10^4^/L and low complement component 4 levels (<5 mg/dL), positive antinuclear antibody (ANA) (1:160 speckled pattern, reference range >1:40), positive anti‐Smith antibody (anti‐Sm), anti‐Ribonucleoprotein (anti‐RNP), double ‐stranded DNA (16 IU/mL, reference range < 12 IU/mL), and anti‐cardiolipin IgG antibody 10 U/mL, confirming the diagnosis of SLE by American College of Rheumatology (ACR) criteria. Lupus anticoagulant and anti‐cardiolipin β2GP1 antibody were negative as part of the screening for antiphospholipid syndrome. A skin biopsy of the erythema on the neck revealed subepidermal cleft with a mixed inflammatory infiltrate of lymphocytes and eosinophils mostly localized in a superficial dermis: occasional vacuolar interface changes and perivascular lymphocytic inflammation were observed (Figure 1b). Direct immunofluorescence (DIF) showed granular deposition of IgM and C3 at the basement membrane zone (BMZ) (Figure 1c and d), a typical histological picture of cutaneous LE. Indirect immunofluorescence (IIF) was negative. Anti‐BP180 Ab was not detected. Urinalysis showed trace protein without microscopic hematuria, pyuria, or urinary casts. The median Systemic Lupus Erythematosus Disease Activity Index (SLEDAI) score at the time of cutaneous SLE diagnosis was 9, suggesting the presence of moderate or severe SLE disease in the patient. Because a recent study in a lupus cohort showed that 11.4% of patients had a disease exacerbation after SARS‐CoV‐2 vaccination,^7^ we were concerned about exacerbation of SLE or onset of adverse events of the vaccine. Because there was no signs or symptoms of active lupus at that time, the patient received the first dose of SARS‐CoV‐2 mRNA vaccine (Pfizer–BioNTech). Fourteen days after receiving the first dose of the SARS‐CoV‐2 mRNA vaccine, he showed deterioration of SLE. Systemic corticosteroid, 20 mg of prednisone was started with significant improvement (Figure 2). On day 40 after the second vaccination, he developed multiple blisters without erythema on the neck and arms (Figure 3a). He had been treated with Teneligliptin for 6 years without any adverse reactions. The skin biopsies obtained from the bullous lesions showed subepidermal bulla with a lymphocyte infiltrate (Figure 3b). DIF of the lesions showed a deposition of IgG on the BMZ (Figure 3c). IgG was positive at the epidermal sides of 1 M NaCl salt‐split skin in indirect immunofluorescence test (Figure 3d). The patient's serum was negative for IgG antibodies against BP180NC16a domain and collagen VII, but positive for full‐length BP 180 antibody (178.9 index value, ELISA). Teneligliptin was withdrawn with improvement. Four weeks later, low C4 levels increased and a white blood cell count increased to 7100/μL. Hydroxychloroquine was discontinued with complete resolution of the bullae. Prednisone dose was tapered to 10 mg daily without BP flare. Figure 2 shows changes of the disease course of the patient.

## 3 DISCUSSION

The clinical diagnosis of early lesions or progression to severe disease in SLE, even if coupled with laboratory findings, remains a challenge given its marked heterogeneity in presentation and clinical progression. Among them, bullous SLE can be mostly and easily confused with other subepidermal bullous diseases^8^ such as BP and bullous drug eruption. The immunopathology findings in bullous SLE include DIF with linear IgG deposition at the BMZ and salt‐split skin indirect IF revealed IgG deposition at the dermal side of the cleavage, findings highly suggestive of bullous SLE.^9^ Because the patient had shown IgM but not IgG deposition at basement membrane with subepidermal cleft, he was not given the diagnosis of bullous SLE before SARS‐CoV‐2 vaccination. However, all findings suggestive of the diagnosis of bullous SLE after SARS‐CoV‐2 vaccination. In this case, SLE controlled by low dose prednisone (20 mg/day) was converted to DPP‐4i‐BP upon SARS‐CoV‐2 mRNA vaccination. Because there have been reported cases of SLE development following SARS‐CoV‐2 mRNA vaccination,^10^ exacerbations of SLE after mRNA vaccination was a sufficient reason of concern. Unpredictably, however, the post‐vaccine conversion to DPP‐4i‐BP occurred 40 days after the second SARS‐CoV‐2 mRNA vaccination. Importantly, despite the prolonged administration of a well‐identified iatrogenic risk factor for the BP, namely DPP‐4i, during the pre‐vaccine period, this patient did not develop DPP‐4i‐BP. In view of the recent finding that SARS‐CoV‐2 mRNA vaccine increases the levels of type I IFN, whose activation has been suggested to play in important role in the pathogenesis of SLE,^11^ a reasonable explanation for this conversion would be that SARS‐CoV‐2 mRNA vaccine may paradoxically serve to protect against SLE flares or to unmask new BP antigens that results in the production of auto‐Abs against self‐antigens, such as BP 180 antigen. Alternatively, IL‐6, IL‐10 and G‐CSF shown to be produced after SARS‐CoV‐2 mRNA vaccines would drive immune responses toward the Th2 functional phenotype, BP. Considering the increase in C4 levels after vaccination, the mRNA vaccines may have served to convert the clinical phenotype to Th2‐mediated autoimmune disease in individuals already predisposed toward the excessive type I IFN production which manifested SLE.

The limitation of our study includes unavailability of important information on the comparisons of cytokine levels and clinical parameters, for example ferritin and D‐dimer before and after SARS‐CoV‐2 mRNA vaccination. SARS‐CoV‐2 vaccine‐induced DPP‐4i‐BP could result from vaccine‐mediated activation of pro‐inflammatory pathways including excessive production of type I IFN^12^ via binding to Toll‐like receptors (TLR3, TLR7, and TLR8). Because the causative drug (DPP‐4i) had been introduced in our patients in the 4 months preceding BP onset, pre‐existent, subclinical autoreactive immune responses against BP180 antigens would be unmasked by vaccine‐induced activation of pro‐inflammatory pathways.^13^, ^14^ Indeed, auto‐Abs against BP 180 was not unmasked even by vaccine‐induced activation of pro‐inflammatory pathways. In our patient, hydroxychloroquine was added 2 weeks after SARS‐CoV‐2 vaccination for bullous SLE with further improvement of decreased C3 and C4 levels and skin lesions. Therefore, it is unclear if hydroxychloroquine added an extra benefit to improvement of SLE. Consistent with this possibility, type I IFN has been shown to induce the maturation and activation of myeloid dendritic cells and promote B cell survival and differentiation into antibody‐producing cells.^15^ These potential insights deserve further investigation.

This is the first case reported in the literature that describes a patient with bullous SLE converted to DPP‐4i BP after COVID‐19 vaccine. Despite long‐term administration of DPP‐4i, this patient did not develop DPP‐4i‐BP during the pre‐vaccine period but developed DPP‐4i‐BP after vaccine. This case highlights the importance of recognition of the vaccine‐ induced conversion from SLE to BP.

## FUNDING INFORMATION

This study was supported by Kawasaki Medical School, a grant from the Japan Agency for Medical Research and Development (20ek0410068h0001), and a Ministry of Health, Labour and Welfare (MHLW) Health and Labor Sciences Research Grant for the Research on Rare Intractable Dermatological Disorders as part of the MHLW Research Project on Overcoming Intractable Diseases.

## CONFLICT OF INTEREST

None declared.
